# SARS-CoV-2 Poorly Replicates in Cells of the Human Blood-Brain Barrier Without Associated Deleterious Effects

**DOI:** 10.3389/fimmu.2021.697329

**Published:** 2021-07-27

**Authors:** Orianne Constant, Jonathan Barthelemy, Karine Bolloré, Edouard Tuaillon, Fabien Gosselet, Christine Chable-Bessia, Peggy Merida, Delphine Muriaux, Philippe Van de Perre, Sara Salinas, Yannick Simonin

**Affiliations:** ^1^Pathogenesis and Control of Chronic and Emerging Infections, University of Montpellier, INSERM, EFS, Antilles University, Montpellier, France; ^2^Univ. Artois, UR 2465, Laboratoire de la Barrière Hémato-Encéphalique (LBHE), Lens, France; ^3^Centre d’Etude des Maladies Infectieuses et de Pharmacologie Anti-Infectieuses, CNRS, Université de Montpellier, Montpellier, France; ^4^Institut de Recherche en Infectiologie de Montpellier, CNRS, Université de Montpellier, Montpellier, France; ^5^Laboratory of Virology, Centre Hospitalier Universitaire de Montpellier, Montpellier, France

**Keywords:** SARS-CoV-2, COVID-19, central nervous system, blood-brain barrier, inflammation

## Abstract

Various neurological symptoms have been associated to severe acute respiratory syndrome coronavirus 2 (SARS-CoV-2) infection including headache, fever, anosmia, ageusia, but also, encephalitis, Guillain-Barre syndrome and ischemic stroke. Responsible for the current coronavirus disease (COVID-19) pandemic, SARS-CoV-2 may access and affect the central nervous system (CNS) by several pathways such as axonal retrograde transport or through interaction with the blood-brain barrier (BBB) or blood-cerebrospinal fluid (CSF) barrier. Here, we explored the molecular and cellular effects of direct SARS-CoV-2 infection of human BBB cells. We observed low replication of SARS-CoV-2 that was accompanied by very moderate inflammatory response. Using a human *in vitro* BBB model, we also described low replication levels without strong inflammatory response or modulation of endothelium integrity. Finally, using serum samples from COVID-19 patients, we highlighted strong concentrations of pro-inflammatory factors that did not perturb BBB integrity after short term exposure. Altogether, our results show that the main mechanism of brain access following SARS-CoV-2 infection does not seem to be directed by brain infection through endothelial cells.

## Introduction

On December 2019, 31th, the World Health Organization (WHO) was informed of an atypical case of pneumonia with unknown origin in Wuhan, Hubei region, China (1). This was the official beginning of the current pandemic of coronavirus disease 2019 (COVID-19) caused by a novel human β-coronavirus and that led to more than 2 800 000 deaths worldwide to date (April 2021). The severe acute respiratory syndrome coronavirus 2 (SARS-CoV-2) is closely related to SARS-CoV, responsible of the 2002-2003 epidemic, mainly in China (2). They are both single-stranded RNA viruses and share the same major human receptor, the angiotensin converting enzyme 2 (ACE-2) (2, 3). SARS-CoV-2 infection can be asymptomatic or cause mild to moderate disease such as fever and respiratory disorders, or serious acute respiratory distress syndrome (ARDS) that can be associated with death (4). COVID-19 symptomatology, in particular for severe cases, is mainly due to immune system dysregulation that lead to cytokine storm and strong deleterious effects on organs (5, 6).

Others common symptoms include fatigue, dry cough, diarrhea or conjunctivitis. Importantly, many patients developed also neurological impairments including nausea, vomiting, anosmia, ageusia, headache, dizziness, encephalitis, meningoencephalitis, Guillain-Barre syndrome, epileptic seizures, ischemic and hemorrhagic stroke. The mechanisms of neuroinfection and potential direct effects of SARS-CoV-2 in the central nervous system (CNS) are still poorly characterized (7–11). During the two last coronavirus epidemics, SARS-CoV in 2002-2003 and the Middle East respiratory syndrome coronavirus (MERS-CoV) in 2012, neurological manifestations were also reported (12). The presence of SARS-CoV was detected in the brain and cerebrospinal fluid (CSF) of deceased patient (13), whereas 4 patients infected by MERS-CoV developed Bickerstaff’s encephalitis and Guillain-Barre syndrome, without any associated respiratory symptoms (14). Recent studies in COVID-19 patients have described the presence of viral RNA in brain tissues (15–17) and viral proteins in endothelial cells of the olfactory bulb (18) or in the CNS (17). Thus, suggestions of direct CNS infection by SARS-CoV-2 have been proposed especially since studies have shown susceptibility to infection of various models *in vivo* and *in vitro* (mouse models, organoids, induced pluripotent stem cell-derived neurons) (19–22). Furthermore, pathological effects on the blood brain barrier (BBB) of the viral protein spike were suggested, that could be consistent with a direct effect of the virus on the BBB homeostasis (23, 24). Notably, in SARS-CoV-2 infections, intracerebral hemorrhages have been reported (25, 26), indicating the negative impact of the virus at the cerebrovasculature (27). Recent evidence indicate a thromboembolic risk in COVID-19 patients (28–31) and the hypothesis of BBB involvement during SARS-CoV-2 infections is clearly emerging (31).

Viruses can reach the CNS by several mechanisms such as axonal transport, direct infection of brain microvascular endothelial cells, transcytosis or by the “Trojan horse” mechanism using infected immune cells from the systemic circulation that can naturally cross brain barriers (32–34). After viral infection, the systematic production of inflammatory cytokines can temporally cause BBB dysregulation, which, in some case, allows further viral entry and facilitates CNS invasion (35).

In this study, we aimed to monitor potential direct SARS-CoV-2 infection of BBB cells and the permissivity of a human *in vitro* model of BBB to infection and systemic inflammatory environment. We employed a cellular BBB model using human brain-like endothelial cells (hBLECs) differentiated from hematopoietic stem cells, presenting the main characteristics of the human BBB (36–39) and previously used to explore other virus infection and replication mechanisms (40–42). Finally, we investigated the role of the inflammatory environment in plasma of COVID-19 patients on the BBB integrity. Investigating the mechanisms used by SARS-CoV-2 to cause neurological impairments and highlighting the neuropathogenesis of this new human β-coronavirus will allow deeper knowledge on brain viral infection, better COVID-19 patient care and possibility to help in the global research effort.

## Materials and Methods

### Cell Culture

Vero E6 cells (African green monkey kidney cells) were obtained from ECACC and maintained in complete Dulbecco’s modified Eagle medium (DMEM) (Pan Biotech) containing 10% fetal bovine serum (FBS), 25 mM HEPES buffer, 100 µg/mL streptomycin and 100 U penicillin. Huh7 (hepatocellular adenocarcinoma) were grown in the same complete DMEM without HEPES buffer. Human pericytes (human brain vascular pericytes, catalog #1200, ScienCell) and astrocytes (astrocytes, catalog #1830, ScienCell) were maintained on poly-l-lysine-coated plates whereas human cerebral endothelial cells (CECs (human brain microvascular endothelial cells), catalog #1000, ScienCell) were grown on fibronectin-coated plates and cultured according to the manufacturer’s instructions.

### Virus Strain and Cellular Infection

The strain BetaCoV/France/IDF0372/2020 was supplied by the National Reference Centre for Respiratory Viruses hosted by Institut Pasteur (Paris, France) and headed by Dr. Sylvie van der Werf. The human sample from which strain BetaCoV/France/IDF0372/2020 was isolated has been provided by Dr. X. Lescure and Pr. Y. Yazdanpanah from the Bichat Hospital. Moreover, the strain BetaCoV/France/IDF0372/2020 was supplied through the European Virus Archive goes Global (Evag) platform (European Union’s Horizon 2020 research and innovation program grant agreement No 653316). The virus was amplified on 80% confluent Vero E6 cells at MOI 0.01; supernatant was collected 72 hours later; samples were collected after centrifugation at 250*g* to remove cellular debris and kept at -80°C. Viral titers were determined using the Spearman-Kärber method and expressed as 50% tissue culture infective dose per milliliter (TCID50/mL).

All infections were done on 80% confluent cell culture, at MOI 1 or 10, in reduced volume of medium during 2 hours at 37°C with permanent agitation before removing inoculum. For each experiments, triplicates were achieved. Supernatants of all experiments were collected at 2, 6 and 10 dpi. Viral replication in each cellular types was determined using the Spearman-Kärber method on previously collected supernatants and expressed as 50% tissue culture infective dose per milliliter (TCID50/mL) as previously published (43).

### Human Blood-Brain Barrier Model and Permeability Tests

**Figure 1:**
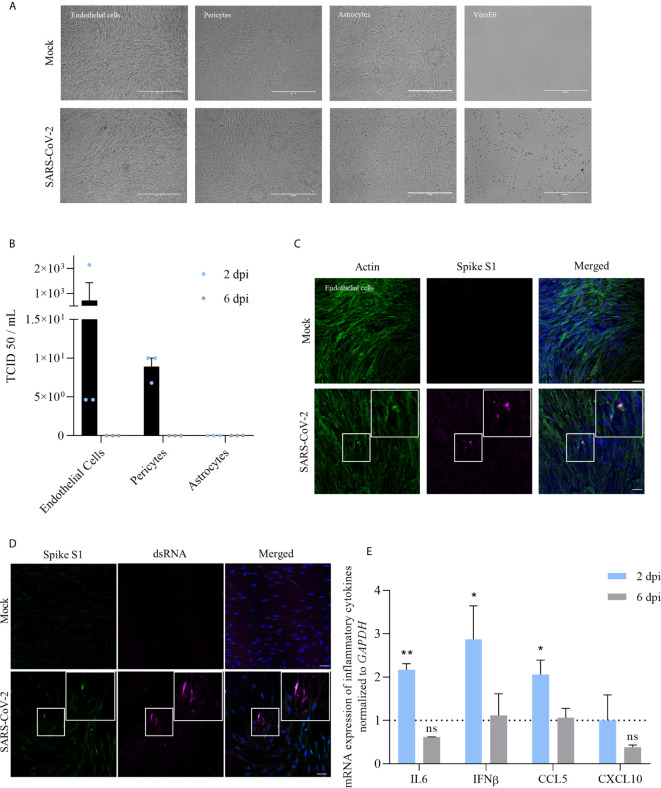
Infection of the BBB cells by SARS-CoV-2 shows a limited replication and induction of cytokine response at MOI of 10. **(A)** Images of the different human BBB cells and Vero E6 infected by SARS-CoV-2 or mock-infected after 2 dpi at MOI 1. **(B)** TCID50 measurements on Vero E6 cells of supernatants of different BBB cells 2 and 6 dpi (MOI 1). Results are expressed as mean ± SEM. **(C)** Immunofluorescence staining of spike subunit S1 in CEC at MOI 10 shows strong staining is sparse cells and diffuse staining that could represent basal replication. Scale bar = 50 µm. **(D)** Immunofluorescence staining of dsRNA in CEC at MOI 10. Scale bar = 50 µm. **(E)** RT-qPCR analyses of *IL-6, IFN-β, CCL5* and *CXCL10* mRNA expression after 2 or 6 dpi from human brain endothelial cells infected by SARS-CoV-2 at MOI 10. Results are expressed as means ± SEM of fold change regulation compared to non-infected cells (n = 6, ns, non significant, *p < 0.05 **p < 0.01).

As previously published (40), the *in vitro* human BBB model (**Figure 1E**) is based on CD34^+^ blood-derived endothelial cells cultured on Matrigel-coated Transwell filters (Costar, 0.4 µm). Collection of human umbilical cord blood was done after infants’ parents signed consent form in compliance with French legislation. The cultivation protocol was approved by the French Ministry of Higher Education and Research (CODECOH Number DC2011-1321), and all experiments were carried out in accordance with the approved protocol.

Filters are placed on top of bovine brain pericytes in 12-well plates, which lead after differentiation to human brain-like endothelial cells (hBLECs) that reproduce the main characteristics of the human BBB in terms of tight-junctions and specific transporters expression (36, 44). Medium was changed every 2 days for 6 days and then endothelial permeability (Pe) was measured with Lucifer Yellow (LY) (20µM; Life Technologies). In physiological conditions, LY is a small hydrophilic molecule presenting a low cerebral penetration. Briefly, Pe was calculated after 1 hour of LY paracellular passage by detection of fluorescence performed on a Tecan SPARK 10M machine with 432/538 nm of excitation/emission wavelength (nm) settings. Endothelium was considered as impermeable when Pe < 1 x 10^-3^ cm/min according to the protocol (37). Viral infection was performed 5 to 6 days after system establishment at MOI 1 and then supernatants were collected at 2, 6 and 10 dpi. As mentioned above, viral replication was determined using the Spearman-Kärber method. For the plasma study, 50 µL of pure plasma sample (7 plasmas from COVID-19 patients and 5 healthy control plasma; all selected randomly) were added directly on the BBB in 300 µL of media for 48 hours. In all the cases (after 6 or 10 days for viral infection; 2 or 24 hours after spike incubation; after 48 hours of plasma incubation), permeability was assessed as described here.

Permeability was also estimated by the measure of transendothelial electric resistance (TEER) using the Epithelial Volt/Ohm Meter EVOM2 (World Precision Instruments, Hertfordshire, UK) according to the manufacturer’s instructions. Briefly, electrodes were sterilized in 70% ethanol for 5 min, rinsed in MilliQ water and equilibrated in BBB media. Then, electrodes were placed in the compartmentalized chambers with the longer electrode vertically touching the bottom of the dish in the lower chamber and the shorter electrode in the upper chamber without touching the cell layer. Values of TEER was recorded once stabilized and to calculate the final TEER values (Ohms.cm^2^), a background measurement of Matrigel (coated insert without cells) was subtracted from the reading and the value was multiplied by the growth surface area.

### Virus Binding Assay

Endothelial cells were placed on fibronectin-coated 24-wells plates and incubated with goat anti-ACE-2 IgG [15µg/mL, described to block receptor-ligand interaction by Hoffman et al. (45)] during 1 hour at 37°C before infection at MOI 10 during 1h at 4°C. Inoculum was removed after infection and supernatants were collected 2 dpi for TCID50 assay.

### Western Blot

Human pericytes, endothelial cells and Vero E6 cells were cultured for 48 h. Then, cells were washed twice in PBS, directly lysed with RIPA. Total protein concentration was calculated using a Bradford protein assay kit. 17µg of total cell lysates were diluted in Laemmli buffer and proteins were separated by SDS-PAGE on 8% (for human Ace2-protein) acrylamide gels. Gels were transferred to PVDF membrane using wet transfer with Tris-glycine-methanol buffer. Membranes were washed in TBS, blocked with 5% milk in TBS-Tween for 30min and incubated overnight at 4°C with primary antibody: anti-human ACE2 (Santa cruz, cat# sc73668), diluted at 1:500 in TBS-T with 5% milk. After washing with 5% milk in TBS-T, the membranes were incubated with Horse-Radish Peroxidase (HRP) conjugated anti-mouse antibodies overnight at 4°C, and with HRP conjugated anti-mouse antibody for 2h at room temperature, washed again in TBS-T, incubated with ECL reagent (Amersham cat#RPN2236and RPN2235) and imaged using a Chemidoc Imager (Biorad). Membrane blotted with anti-hACE2 were incubated with anti-HRP-conjugated GAPDH (Sigma, cat# G9295).

### Materials

Antibodies used are the follows: anti-SARS-CoV-2 spike glycoprotein (272504, Abcam), rabbit anti-ZO1 (617300; Invitrogen), mouse anti-dsRNA (J2; Scicons), goat anti-ACE-2 (AF933, R&D systems), ActinGreen (R37110, Thermo Fisher Scientific), Hoescht (purchased from Sigma).

### Spike Protein S1 Subunit Test

The recombinant human coronavirus SARS-CoV-2 spike glycoprotein S1 (ab273068, Abcam) was incubated on hBLECs at 10 nM during 2 or 24 hours. Then supernatants and RNA were collected, permeability assay was achieved according to precedent detailed method and transwell inserts were also fixed in 4% PFA after one rinse in phosphate-buffered saline (PBS).

### Immunofluorescence Assay

For specific immunofluorescence assays, endothelial cells were plated on fibronectin-coated coverslips and Huh7 directly on coverslips. After recovering supernatants, coverslips or transwell filters were rinsed with PBS. Cells were fixed in 4% PFA during 15 min at room temperature (RT) and permeabilized with 0.1% Triton X-100/PBS for 5 min at RT. Then, they were blocked for 30 min to 1 hour in 2% bovine serum albumin (BSA), follow by incubation in primary and secondary antibodies before incubation with Hoescht (Sigma) and assembling in fluorescent mounting medium (Prolongold, Thermo Fisher). Cells were imaged by confocal microscopy with the Zeiss SP85 confocal microscope, using 40x or 63x 1.4 NA Plan Apochromat oil-immersion objectives.

### RT-qPCR

Mock- and SARS-CoV-2- infected cells were lysed in RLT buffer (Qiagen), then, RNA were extracted with RNeasy Mini-Kit (Qiagen). From these, cDNA were synthesized using reverse transcription (Omniscript reverse transcriptase, Qiagen) and analyzed on a LightCycler 480 real-time PCR apparatus (Roche). Viral RNA from mock- and SARS-CoV-2-infected supernatants were extracted using the Qiamp viral RNA Mini Kit (Qiagen). GAPDH or HPRT1 gene was used to normalize samples.

Sequences of human primers used for amplification are the followings: for ACE-2: 5’-GGACCCAGGAAATGTTCAGA (fwd) and 5’-GGCTGCAGAAAGTGACATGA (rev); for CCL5: 5’-ACT GCCCCGTGCCACATCAA (fwd) and 5’-CTGGGTTGGCACACACTTGGC (rev); for Claudin-5: 5’-TTAACAGACGGAATGAAGTT (fwd) and 5’-AAGCGAAA TCCTCAGTCT (rev); for CXCL10: 5’-TATTCCTGCAAGCCA ATTTTGTC (fwd) and 5’-TCTTGATCGCCTTCGATTCTG (rev); for GAPDH: 5’-CATTGACCTCAACTACATGC (fwd) and 5’-GCCATGCCAGTGAGCTTCC (rev); for HPRT1: 5'-AGCTTGCTGGTGAAAAGGAC (fwd) and 5'-TTTATAGTCAAGGGCATATCC (rev); for IFN-β: 5’-GTC TCCTCCAAATTGCTCTC (fwd) and 5’-ACAGGAGCTTCT GACACTGA (rev); for IL-6: 5’-CCAGGAGCCCAGCTAT GAAC (fwd) and 5’-CCCAGGGAGAAGGCAACTG (rev); for Occludin: 5’-TTCTGGATCTCTATATGGTTCA (fwd) and 5’-CCACAACACAGTAGTGATAC (rev); for Z0-1: 5’-CCTGA ACCAGTATCTGATAA (fwd) and 5’-AATCTTCTCACTC CTTCTG (rev); for TMPRSS2: 5’-GAGGTGAAAGCGGGTG TGAG (fwd) and 5’-ATAGCTGGTGGTGACCCTGA (rev); for CTSL: 5’-CTGGTGGTTGGCTACGGATT (fwd) and 5’-CTCC GGTCTTTGGCCATCTT (rev).

### COVID-19 Patient Plasma

Plasmas were collected thanks to the virology unit of the Lapeyronie hospital in Montpellier. They were given by different units for the COVID-19 patient plasma (n = 10) (**Supplementary Table 1**) and by the occupational healthcare for the negative controls (n = 10). Until use, samples were stored at -80°C, then aliquots were done on ice and were directly used for cytokine measurement by multiplex ELISA or for the BBB permeability assay.

### Multiplex Assay

A multiplex bead-based assay for the quantification of 20 human inflammatory cytokines (ProcartaPlex Human Inflammation Panel 20plex, Thermo Fisher Scientific) was performed according to the manufacturer procedures and recorded using a Luminex apparatus (MAGPIX; Thermo Fisher Scientific). Plasma samples were used to analyze a cytokine panel representing innate immune response against viral infection. In healthy controls (*n* = 10) and in COVID-19 samples (*n* = 10), the cytokine concentrations detected were reported in pg/mL.

### Enzyme-Linked Immunosorbent Assay (ELISA)

Plasma of COVID-19 and healthy patients were used to perform an ELISA assay for human ICAM (R&D systems). Analyze was done on a spectrophotometer by measurement of absorbance at 450 nm (Thermo Fischer Scientific).

### Statistical Analysis

For all statistical analysis, a minimum of three independent experiments were done and unpaired Mann-Whitney tests were performed using Prism software.

### Ethics Statement

All adult subjects provided written informed consent, and a parent or guardian of any child participant provided informed consent on the child’s behalf, in compliance with the French legislation. For human BBB model the protocol was approved by the French Ministry of Higher Education and Research (CODECOH Number DC2011-1321).

## Results

### SARS-CoV-2 Shows Moderate Replication in the BBB Cells With Low Associated-Inflammatory Response

To better understand the ability of SARS-CoV-2 to infect cells of the BBB, we performed infection of primary human cerebral endothelial cells (CECs), pericytes and astrocytes at a multiplicity of infection (MOI) of 1. No cytopathogenic effect (CPE) was observed after infection in these cells contrary to Vero E6 infected cells (**Figure 1A**). At 2 days post-infection (dpi), we observed no replication (determined by TCID50/ml assay) in astrocytes and low replication in pericytes and CECs. Viral replication in human pericytes and CECs appeared to be transient, as we could not detect viral titer at 6 dpi (**Figure 1B**). In the same conditions, we found a greater replication in Huh7 cells, a permanent cell line established from human hepatoma tissue previously described to be permissive to SARS-CoV-2 infection (~ 10^5^ TCID50/mL at 2 dpi, data not shown) (46).

At 2 dpi, infected-CECs were fixed and, using an immunofluorescence approach, stained with a specific antibody against SARS-CoV-2 spike protein S1 subunit (**Figure 1C**) and an anti-dsRNA antibody (**Figure 1D**). Notably, we showed the presence of dsRNA in CECs, consistent with active replication of SARS-CoV-2 (**Figure 1D**). We then performed RT-qPCR studies of infected-CECs and pericytes at 2 and 6 dpi and assayed the expression of inflammatory molecules and did not detect significant modulation in the expression of *IL-6*, *IFN-β*, *CCL5*, or *CXCL10* mRNAs compared to mock-infected cells (**Supplementary Figure 1**). Of note, we did not observe significant higher replication rates at higher MOI (MOI of 10, ~ 1.10^3^ TCID50/ml at 2 dpi) but we detected a significant increase of *IL-6*, *IFN-β* and *CCL5* mRNA expression at 2 dpi in CECs (**Figure 1E**).

### SARS-CoV-2 Poorly Replicates in a Human *In Vitro* BBB Model Without Permeability Alteration or Strong Associated-Inflammatory Response

**Figure 2:**
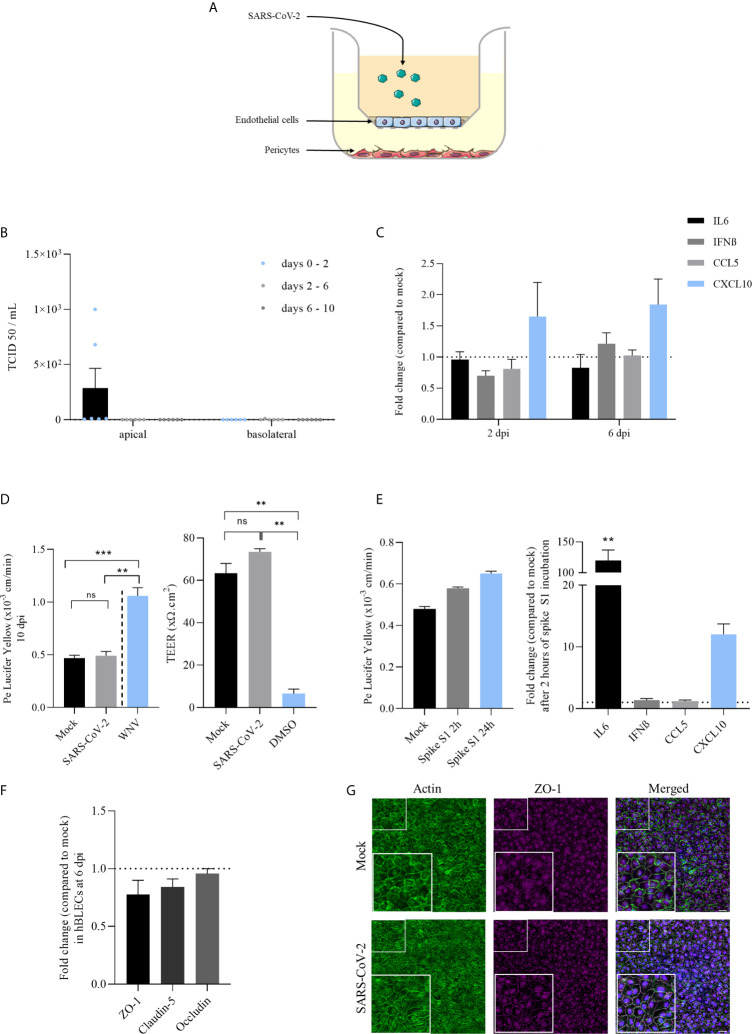
Infection of a human *in vitro* BBB model by SARS-CoV-2 reveals weak replication, limited induction of inflammatory cytokines and did not perturb the specific brain endothelial structure. **(A)** The *in vitro* BBB model is composed of primary brain pericytes cultured at the bottom of plastic plates, allowing the differentiation of CD34^+^ blood cord-derived endothelial cells into human brain-like endothelial cells (hBLECs) on transwell filters in the apical chamber. Virus is added in this compartment representing the blood side of the BBB. **(B)** Replication of SARS-CoV-2 in hBLECs in apical and basolateral compartments at 2, 6 and 10 dpi at MOI 1, titrated by TCID50 measurement on Vero cells. Results are expressed as mean ± SEM. **(C)** Inflammatory responses following SARS-CoV-2 infection of hBLECs at MOI 1 expressed in mRNA fold change compared to non-infected cells at 2 and 6 dpi. **(D)** Permeability coefficient of Lucifer Yellow 10 days after infection of hBLECs by SARS-CoV-2 or WNV at MOI 1. Bars show mean of 3 independent experiments ± SEM (n = 6; ns = non significant, **p < 0.01 ***p < 0.001); Transendothelial electric resistance (TEER) measures done after 10 dpi on mock- and infected-hBLECs. Positive control of permeable BBB was used by adding 20% DMSO during one hour before measurement. Bars show mean of 3 independent experiments ± SEM (n = 6; **p < 0.01). **(E)** Permeability coefficient of Lucifer Yellow following 2 or 24 hours after incubation of hBLECs with 10 nM of spike S1 subunit recombinant protein. Bars show mean of 3 independent experiments ± SEM; Inflammatory responses following spike S1 subunit recombinant protein incubation of hBLECs at 10 nM expressed in mRNA fold change compared to non-incubated cells 2 hours after (n = 6; **p < 0.01). **(F)** Tight junction protein mRNA expression following SARS-CoV-2 infection of hBLECs at MOI 1 compared to mock at 6 dpi. **(G)** Immunofluorescence staining of ZO-1 and actin in hBLECs at 10 dpi. Scale bar = 50 µm.

As mentioned above, the BBB is a very important structure to control pathogen CNS access. To investigate whether SARS-CoV-2 is able to directly infect or cross the BBB, we used a human BBB model that recapitulates the main characteristics of the barrier (36, 39). This cellular model is composed of endothelial cells differentiated from CD34^+^ cord blood-derived hematopoietic stem cells cultured on transwell filter inserts that further differentiate in brain-like endothelial cells (hBLECs) in the presence of brain pericytes (36) (**Figure 2A**). Similarly, that what happens *in vivo*, pericytes will induce and maintain BBB properties by secreting diffusible factors allowing the differentiation of the endothelial cells into BBB endothelial cells. To study viral replication and inflammatory pathways modulation, supernatants and mRNAs were collected at 2, 6 and 10 dpi from mock- or infected-hBLECs and pericytes (MOI 1). TCID50 measurements showed very low titers in hBLECs at 2 dpi (≥ 2,6.10^2^ TCID50/mL, corresponding to the production between 0 and 2 days) that decreased at 6 dpi (production between 2 and 6 days) and 10 dpi (production between 6 and 10 days) (**Figure 2B**). In the basolateral compartment, no significant replication was observed (**Figure 2B**). We also assessed whether pericytes from the BBB model were susceptible to the SARS-CoV-2 infection. Similarly to primary human pericyte culture, no CPE (**Supplementary Figure 2A**) and only weak and transient replication were detected in infected cells (**Supplementary Figure 2B**). We next monitored inflammatory response factors in infected cells. At 2 and 6 dpi, mRNA expressions of *IL-6*, *IFN-β*, *CCL5* and *CXCL10* were measured and only *CXCL10* was slightly increased (but not significantly) in infected hBLECs (**Figure 2C**).

We then analyzed the effect of SARS-Cov-2 infection on BBB integrity using a fluorescent-based assay with Lucifer Yellow (LY) and by the measurement of the transendothelial electric resistance (TEER). We did not notice significant change of Pe (permeability coefficient, see *Material and Methods*) value at 10 dpi, as we detected a value of ~ 0.5x10^-3^ cm/min, for both mock and infected-BBB model, consistent with a “tight” BBB endothelium (36) (**Figure 2D**). In comparison, we performed an infection with West Nile virus (WNV, MOI 1) described for its ability to induce disruption of BBB integrity (47), where we obtained a Pe above 1x10^-3^ cm/min, thus reflecting a disturbed impaired endothelium (**Figure 2D**). Similarly, we did not measure significant change in TEER measures at 10 dpi for both mock- and infected-hBLECs, while the DMSO led to significant loss of TEER consistent with a strong disruption of the BBB endothelium (**Figure 2D**). Previous studies described the capacity of spike S1 subunit from SARS-CoV-2 envelope to cross the BBB and alter baseline permeability (23, 24). Therefore, we investigated the effect of this protein alone in our BBB model. After 2 and 24 hours of incubation with 10 nM of S1, Pe was slightly increased (from 0.48x10-3 cm/min in mock to 0.65x10-3 cm/min after 24 hours of spike incubation) possibly suggesting, in a non-infection context, an effect of high concentration of spike on the BBB integrity (**Figure 2E**). In addition, after 2 hours of incubation, *IL-6* and *CXCL10* mRNA expressions were strongly and significantly increased after spike S1 subunit treatment (**Figure 2E**).

To confirm the absence of effect of SARS-CoV-2 on BBB integrity using another approach, we performed an analysis of tight junction protein mRNA expression at 6 dpi in hBLECs. Normalized to *GAPDH* expression, *ZO-1*, *claudin-5* and *occludin* expressions in infected-cells were not significantly decreased in comparison to mock-infected hBLECs (**Figure 2F**). In parallel, cells were fixed and stained with specific antibodies against ZO-1 and labelled with an actin probe at 10 dpi to monitor endothelium integrity. We observed no difference in the tight junction organization and actin architecture of the endothelium between mock- and SARS-CoV-2- infected hBLECs (**Figure 2G**).

**Figure 3:**
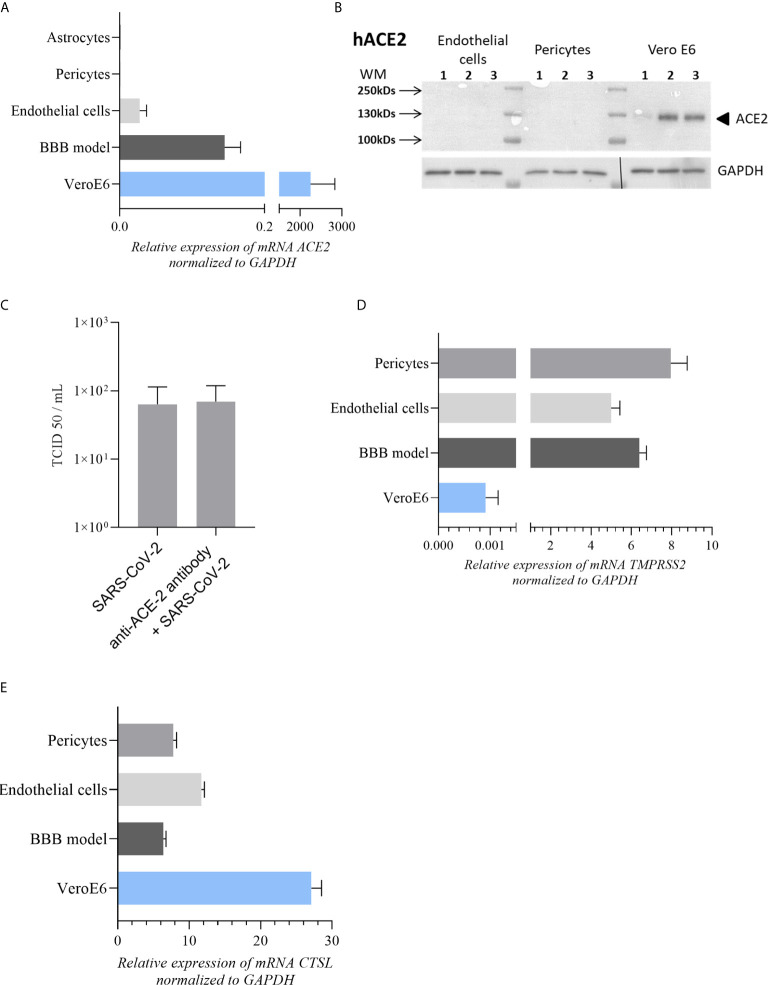
Expression of main SARS-CoV-2 cell receptors in our cell models **(A)** *ACE-2* mRNA expression in astrocytes, pericytes, BBB model and endothelial cells in comparison to Vero E6 cells (highly permissive to SARS-CoV-2 infection). **(B)** No detectable expression of ACE-2 protein by western blot in endothelial and pericytes cells contrary to Vero E6 cells. **(C)** TCID50 measurements of SARS-CoV-2 in CECs at 2 dpi after treatment with anti-ACE-2 antibody. TCID50 measure results are expressed as mean ± SEM. **(D, E)** Respectively *TMPRSS2* and *CTSL* mRNA expression in BBB model, pericytes and endothelial cells in comparison to Vero E6 cells.

Finally, to better understand the low viral replication in BBB cells, we analyzed the expression of hACE-2, which SARS-CoV-2 uses as principal host cell receptor. Here, comparing to VeroE6 cells, we showed a very low expression of *ACE2* gene in CECs and hBLECs as previously described for other human endothelial cells (48) and no detection in primary human pericytes and astrocytes (**Figure 3A**). We also confirmed these observations by analyzing the protein expression level of hACE2 into CECs and pericytes. As showed in **Figure 3B**, while Vero E6 cells express ACE2, we were not able to detect efficiently this protein in CECs and pericytes by Western-Blot. Moreover, by pretreating CECs with a ACE2 blocking-antibody, we did not significantly reduce SARS-CoV-2 replication. Indeed, at to 2 dpi we monitored the replication of SARS-CoV-2 using a TCID50/mL assay, which did not show significant difference, suggesting that this receptor has no major role in BBB SARS-CoV-2 tropism (**Figure 3C**). Then we explored the expression of the co-receptors for SARS-CoV-2 TMPRSS2 (45, 49) and Cathepsin-L (CTSL) (50). We were able to detect expression of *TMPRSS2* (**Figure 3D**) and *CTSL* (**Figure 3E**) mRNA in our cell models that could participate in low viral entry.

### Plasma of COVID-19 Patients Show Upregulated Pro-Inflammatory Cytokines Without Deleterious Effects on Human *In Vitro* BBB

Because SARS-CoV-2 infection did not have obvious effects on BBB homeostasis, we next assayed the effect of the inflammatory environment in COVID-19 patient plasma on brain endothelium using a cohort from the hospital university center of Montpellier. These plasma samples came from persons who developed severe COVID-19 pathology and who have been placed under oxygen therapy after their hospitalization following the occurrence of complications (see **Supplementary Table 1** and *Material and Methods*).

**Figure 4:**
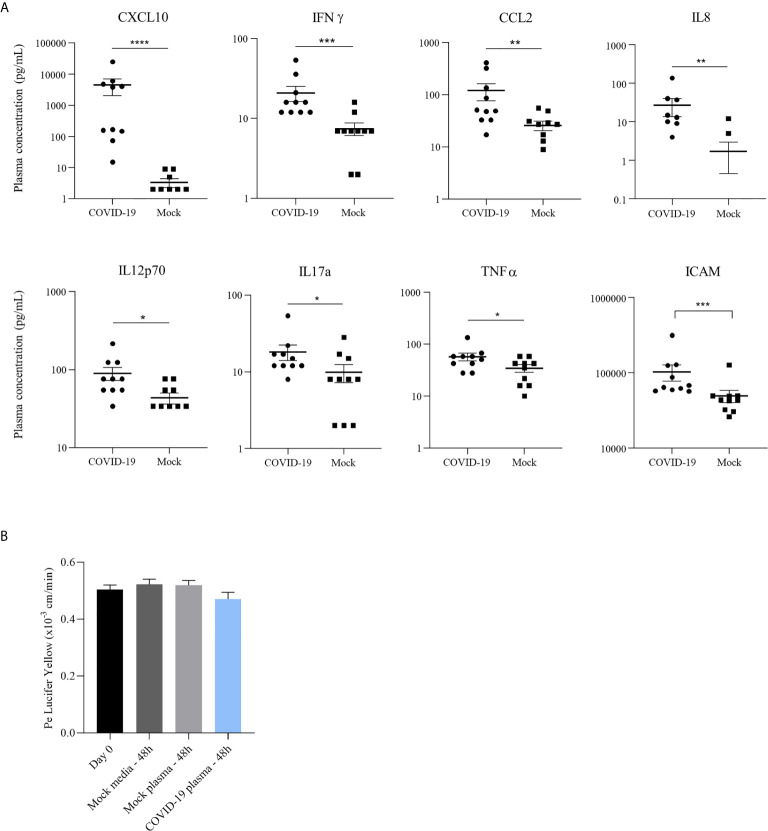
COVID-19 patient plasma do not induce endothelium impairment or inflammatory response in a bi-cultured BBB model despite a pro-inflammatory environment. **(A)** Concentration (pg/mL) of different pro-inflammatory cytokines in plasma of COVID-19 patients hospitalized in Montpellier, France, and non-infected individuals quantified by a multiplex bead-based ELISA: in order, CXCL10, IFN-γ, CCL2, IL-8, IL-12p70, IL-17a and TNF-α; Analyses of soluble ICAM-1 concentration (pg/mL) in plasma from healthy and COVID-19 patients by ELISA assay. Bars show means ± SEM (n = 10; *p < 0.05 **p < 0.01 ***p < 0.001 ****p < 0.0001). **(B)** Permeability coefficient of Lucifer Yellow at day of infection (day 0) and following 48 hours after incubation of hBLECs with different COVID-19 plasmas, healthy control plasma or media. Bars show mean of 3 independent experiments ± SEM.

Inflammatory cytokines were analyzed in the serum by a multiplex assay for the simultaneous quantification of 20 human inflammatory cytokines (ProcartaPlex Human Inflammation Panel 20plex, Thermo Fisher Scientific). Among the 20 inflammatory cytokines measured in the plasma of patient suffering of COVID-19, 8 have shown increased concentration compared to healthy individuals (**Figure 4A**). The strongest upregulation was observed for CXCL10 with a mean of around 4 500 pg/mL in COVID-19 against around 5 pg/mL in healthy sample. We also observed the upregulation of IFN-γ, CCL2, IL-8, IL-12p70, IL-17a and TNF-α in COVID-19 patients compared to healthy controls (**Figure 4A**). Level of soluble ICAM-1, a cell adhesion molecule (CAMs) involved in leukocyte docking to the BBB, was also found statistically modulated in SARS-CoV-2 patients after a specific ELISA assay (**Figure 4A**).

The presence of high cytokine and chemokine concentrations could have a role in vascular endothelium leakage noticed in many case reports (33). We therefore investigated the effect of the inflammatory environment in these plasmas on the human BBB model by incubation of the COVID-19 patient or healthy control samples on hBLECs. The permeability was measured 48h post-incubation and no significant change in Pe was observed (with value ~ 0.5x10^-3^ cm/min, for all conditions tested) (**Figure 4B**). Together, these data indicate a strong inflammatory environment in the blood of SARS-CoV-2 infected patients without direct impairment of the BBB *in vitro* for 48 hours.

## Discussion

The molecular and cellular mechanisms of viral access and neuropathogenesis are of crucial interests to understand physiopathological effects of brain infections. Axonal transport in peripheral and central neurons has been suggested for SARS-CoV-2 (51) similarly than for other neurotropic viruses such as herpes simplex virus (HSV) and WNV (52, 53). Indeed, it has been shown that coronaviruses can infect peripheral neurons from the olfactory bulb that can be gateway for the CNS (54–57). Viruses can also use hematogenous routes for CNS access. Free or cell-associated viral particles in the blood stream can traffic across the BBB and the blood-CSF barrier to gain access to the CNS (32, 58). Direct endothelial cell infection can lead to parenchyma viral release and potential infection of other neuro-vascular cells such as pericytes or astrocytes. Cellular infection can in turn lead to the production of inflammatory mediators that can also modulate endothelium integrity allowing further viral entry. On the other hand, infected-immune cells from the blood can interact with and pass across the BBB to liberate viruses in the CNS, in a mechanism called “Trojan horse” (59).

SARS-CoV-2 infections induce various neurological symptoms and there is now clear evidence that neurological symptoms of COVID-19 are common and in some cases severe (26). Moreover it was recently described that the virus can directly infect neurons and can be found in the brain or in the CSF of COVID-19 patients (15–18). In addition, numerous pathologies affecting neurovasculature have been reported in patients, such as thromboembolism or ischemic stroke, highlighting a potential role of the BBB in the neurological COVID-19 diseases (60, 61). Despite these observations, the mechanisms of SARS-CoV-2 access to the brain are poorly described. One potential SARS-CoV-2 CNS entry mechanism could be by direct endothelial cell infection and viral ability to replicate and to disturb BBB integrity. Viral protein spike was shown to induce perturbation of BBB integrity *in vitro* and to cross the BBB through transcytosis *in vivo* (23, 24). These studies using recombinant S1 protein could recapitulate the effect of viral shed or secreted spike during local replication or in the circulation, albeit very little information exist whether free spike exists in patients. However, the ability of free viral particles of SARS-CoV-2 to cross, disturb or infect the BBB still remains unclear and could differ depending on the system (murine *vs* human for instance). In this study, we showed that SARS-CoV-2 can poorly and transiently infect human brain endothelial cells and pericytes. This is in agreement with others studies that have also shown a very poor infection of endothelial cells notably in human pulmonary (HPAEC) and cardiac (HCAEC) endothelial cells (22, 48). This low infection level could be related to the very low expression of ACE-2, the main SARS-CoV-2 receptor, in endothelial cells. It is difficult to conclude however whether the low replication levels of SARS-CoV-2 is due to low ACE-2 expression level on CECs and pericytes or the presence of alternate, less efficient, receptors or to a post-entry step that would restrict the viral cycle. Our ACE-2 blocking antibody assay did not reduce the low replication, which would favor the hypothesis that albeit very low ACE2 expression levels exists on BBB cells, SARS-CoV-2 may take advantage of other receptors or co-factors, even though only ACE-2 has been identified so far as a cellular receptor (62). Both TRMPSS2 and CTLS proteases are efficiently expressed in BBB cells, and could partially participate in viral entry, as described in several studies (45, 50, 62) but whether they can be involved independently of ACE-2 is not currently known.

However, despite the weak replication level at high MOI, we were able to detect some cytokine response following infection as observed by the expression of IL-6, IFN-β and CCL5 in brain endothelial cells. Our experiments using a human *in vitro* BBB model showed only a very limited replication of SARS-CoV-2 at 2 dpi only. Furthermore, viral replication did not affect the integrity of the endothelium. Similarly to studies where after intranasal infection of mouse models the virus was found in the brain but not in vascular endothelium (22), we demonstrated here that the direct BBB *in vitro* infection does not impact endothelial integrity or the expression of some tight junction proteins (mainly ZO-1). However, SARS-CoV-2 may alter other BBB aspects, for instance the activity of transporters such as efflux pumps that could impair CNS homeostasis. Another way that the virus could affect BBB homeostasis would be from systemic inflammation in response to viral infection that could favor SARS-CoV-2 entry into the CNS, leading to neuroinflammation (63). Indeed host innate responses leading to pro-inflammatory cytokine and chemokine production are thought to modulate BBB integrity and enable viruses to access the brain (64). We, as previous studies, have identified a high amount of inflammatory cytokines in the blood of patients suffering from COVID-19 (5, 65). Here, we did not observe an effect of patient plasma on BBB integrity after 48 hours of exposure. Nonetheless, the increased concentration of these cytokines could have effect on BBB during extended contact (66), especially knowing that this systemic inflammatory state can persist in patients. Inflammation can modulate at long term the BBB integrity and participate in immune cells recruitment which can initiate or amplify neuroinflammation (5, 67). Pro-inflammatory cytokine IL-6 exhibited an increased concentration in blood in COVID-19 patients and could participate in general inflammatory state observed in the disease (5). Of note, IL-6 is also known to modulate BBB integrity by interaction with endothelium structure. Indeed, high concentration of IL-6 in the plasma was correlated with higher BBB permeability and a decrease in tight junction expression mediated by protein kinase C pathway (68–70). Moreover, CCL5 and CXCL10 are chemokines frequently released upon viral infection. CXCL10 also leads to the decrease of tight junction protein expression (71) and can promotes BBB damage by inducing TNFα production (71). Interestingly, increased concentrations of the cell adhesion molecule ICAM-1 in the blood of SARS-CoV-2 infected patients could participate in leukocyte recruitment and exacerbate brain inflammation (72). Strictly, activated leukocytes express integrins, such as MAC-1 or lymphocyte function-associated antigen 1 (LFA-1) for instance, on endothelial cells, major receptors of which are CAMs as ICAM-1 (73). In this way, ICAM-1 can also have a role for “Trojan horse” invasion by facilitating the infiltration of SARS-CoV-2 infected immune cells (74).

As suggested by other studies, SARS-CoV-2 particles can enter the brain and directly induce neurological disorders (21, 67). However, among the proposed CNS mode of access, direct cerebral endothelial cell infection is unlikely to be the major mechanism responsible for SARS-CoV-2 brain infection. It clearly highlights complex mechanisms that could lead to SARS-CoV-2 neurological disease, with implication of several factors such as nerve infection, neurovasculature damages and inflammation. Indeed, the cytokine storm and systemic inflammation associated to COVID-19 remains a key mechanism in the apprehension of all pathologies related to SARS-CoV-2 infection and described during the pandemic.

## Data Availability Statement

The raw data supporting the conclusions of this article will be made available by the authors, without undue reservation.

## Ethics Statement

All adult subjects provided written informed consent, and a parent or guardian of any child participant provided informed consent on the child’s behalf, in compliance with the French legislation. For human BBB model, the protocol was approved by the French Ministry of Higher Education and Research (CODECOH Number DC2011-1321). The patients/participants provided their written informed consent to participate in this study.

## Author Contributions

OC, SS, and YS contributed to conception and design of the study. OC, KB, CC-B, and PM performed the experiments. OC performed the statistical analysis. CC-B produced and titer SARS-CoV-2 stock on Vero E6 cells. DM edited part of the manuscript and raised fundings for SARS-CoV-2 research at Cemipai (CNRS Montpellier). OC wrote the first draft of the manuscript. OC, DM, FG, SS, and YS wrote sections of the manuscript. All authors contributed to the article and approved the submitted version.

## Funding

This work was funded by Montpellier University of Excellence (MUSE) for Cemipai and UMR1058.

## Conflict of Interest

The authors declare that the research was conducted in the absence of any commercial or financial relationships that could be construed as a potential conflict of interest.

## Publisher’s Note

All claims expressed in this article are solely those of the authors and do not necessarily represent those of their affiliated organizations, or those of the publisher, the editors and the reviewers. Any product that may be evaluated in this article, or claim that may be made by its manufacturer, is not guaranteed or endorsed by the publisher.
