# Supplementary figures and images for: SARS-CoV-2 Poorly Replicates in Cells of the Human Blood-Brain Barrier Without Associated Deleterious Effects
Source: Front Immunol. 2021 Jul 27;12:697329. doi: 10.3389/fimmu.2021.697329 (PMC8353323; doi:10.3389/fimmu.2021.697329)

Supplementary figure 1

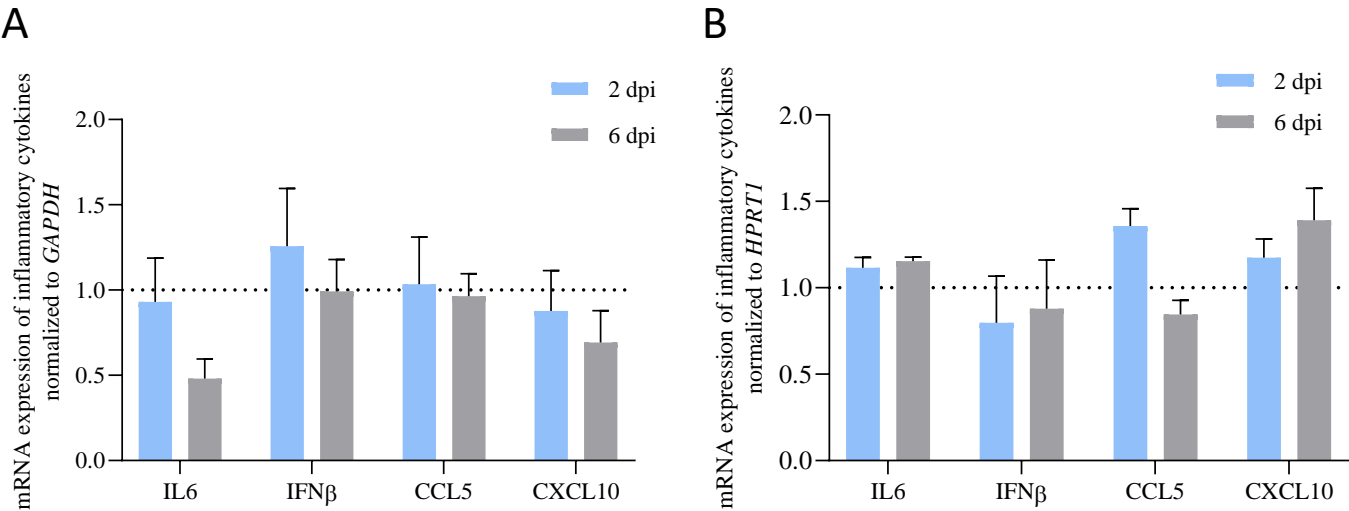

Supplementary figure 2

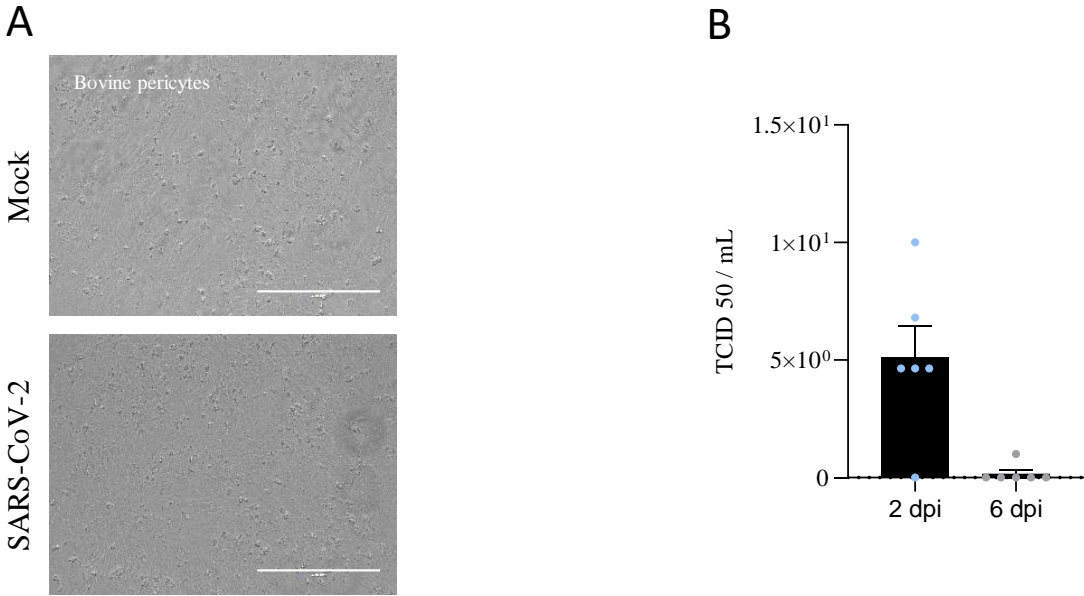

Supplement: Supplementary file 1 [file DataSheet_1.pdf]
