# Supplementary material for: SARS-CoV-2 Poorly Replicates in Cells of the Human Blood-Brain Barrier Without Associated Deleterious Effects
Source: Front Immunol. 2021 Jul 27;12:697329. doi: 10.3389/fimmu.2021.697329 (PMC8353323; doi:10.3389/fimmu.2021.697329)
Supplement: Supplementary file 2 [file Table_1.docx]

| Sexe | Age | Date of 1st symptoms | Date of hospitalization | Collection date |
| --- | --- | --- | --- | --- |
| M | 73 | 10/20/20 | 10/24/20 | 10/24/20 |
| M | 79 | 10/31/20 | 11/04/20 | 11/04/20 |
| M | 60 | 10/26/20 | 11/01/20 | 11/02/20 |
| M | 90 | 11/03/20 | 11/03/20 | 11/04/20 |
| M | 76 | not documented (nd) | 10/29/20 | 10/29/20 |
| M | 76 | 10/17/20 | 10/25/20 | 10/27/20 |
| M | 89 | 10/14/20 | 10/29/20 | 11/03/20 |
| F | 82 | 10/20/20 | 10/26/20 | 10/27/20 |
| M | 62 | 10/21/20 | 10/26/20 | 10/28/20 |
| F | 63 | nd | 10/26/20 | 10/28/20 |

Supplementary Table 1
